# IsoProDB: an integrated map of human protein isoforms for accelerated research

**DOI:** 10.1093/database/baag015

**Published:** 2026-03-26

**Authors:** Sreelakshmi Pathappillil Soman, Samseera Ummar, Muktar Ahmed, Prathik Basthikoppa Shivamurthy, Sourav Sreelan, Poornima Ramesh, Mahammad Nisar, Yashwanth Subbannayya, Rajesh Raju

**Affiliations:** Centre for Integrative Omics Data Science (CIODS), Yenepoya (Deemed to be University), Mangalore 575018, India; Centre for Systems Biology and Molecular Medicine (CSBMM), Yenepoya Research Centre, Yenepoya (Deemed to be University), Mangalore 575018, India; Centre for Integrative Omics Data Science (CIODS), Yenepoya (Deemed to be University), Mangalore 575018, India; Department of Zoology, College of Science, King Saud University, P.O. Box 2455, Riyadh 11451, Kingdom of Saudi Arabia; Centre for Integrative Omics Data Science (CIODS), Yenepoya (Deemed to be University), Mangalore 575018, India; Centre for Integrative Omics Data Science (CIODS), Yenepoya (Deemed to be University), Mangalore 575018, India; Centre for Integrative Omics Data Science (CIODS), Yenepoya (Deemed to be University), Mangalore 575018, India; Centre for Integrative Omics Data Science (CIODS), Yenepoya (Deemed to be University), Mangalore 575018, India; School of Biosciences, Faculty of Health and Medical Sciences, University of Surrey, Guildford GU2 7XH, United Kingdom; Surrey Institute for People-Centred AI, University of Surrey, Guildford, Surrey GU2 7XH, United Kingdom; Centre for Integrative Omics Data Science (CIODS), Yenepoya (Deemed to be University), Mangalore 575018, India; Centre for Systems Biology and Molecular Medicine (CSBMM), Yenepoya Research Centre, Yenepoya (Deemed to be University), Mangalore 575018, India

## Abstract

Emerging studies highlight the importance of protein isoforms, which often exhibit distinct functional roles and contribute to physiological diversity, disease mechanisms, and phenotypic variation, despite originating from the same gene. However, comprehensive isoform-level resources that characterize protein isoforms remain limited. IsoProDB is an integrative and unified one-stop database that aligns protein isoforms from RefSeq and UniProtKB, enabling cross-sequence visualization for protein isoform analysis in humans. It integrates features such as domain architecture, intrinsically disordered regions, sequence variants, transmembrane topology, and 52 distinct post-translational modifications (PTMs) mapped to protein isoforms from multiple resources. Currently, IsoProDB enables users to perform gene wise comparative analyses across 110 149 protein isoforms derived from 20 536 protein-coding genes for all integrated features, supported by effective visualizations. This provides insights into conserved and nonconserved PTM sites, domains, isoform-specific membrane localization, the impact of variants on protein function, and disease relevance across protein isoforms. With specific isoforms emerging as markers and theragnostic targets for various disorders, IsoProDB is integrated with multiple global resources for easy navigation and exploration of multiomics information on isoforms.

**Database URL:**  https://ciods.in/isoprodb

## Introduction

Despite originating from the same gene, protein isoforms have the potential to exhibit distinct biological roles. This has contributed to a growing interest in exploring the structural and functional diversity of proteins at the isoform level. Mechanisms such as alternative splicing, intron retention, and alternative transcription start/stop sites serve to diversify mRNA sequences, yielding different protein isoform [1,2]. In general, it is estimated that >90% of the human genes undergo alternative splicing, and each gene yields, on average, four protein isoforms as reported by previous studies [1,3]. These isoforms often differ in sequence and may have distinct structural and functional properties along with similarities. Moreover, aberrant regulation of protein isoforms has been associated with the development and progression of various diseases. Furthermore, isoforms display functional diversity within these disease contexts. For instance, spleen tyrosine kinase (SYK), a nonreceptor tyrosine kinase, exhibits a dual role in cancer. It acts as an oncogenic driver in leukemia [4], while in certain solid tumours, such as lung and colorectal cancers [5], it promotes cell proliferation, survival, and metastasis. In contrast, SYK acts as a tumour suppressor in breast cancer by inhibiting tumour initiation and progression [6,7]. These opposing functions are largely attributed to changes in the regulation of protein isoforms [8,9]. Moreover, recent studies indicate that the drugs miss their targets when an isoform switch occurs in the synthesis of the target protein. For instance, trastuzumab (a HER2-targeted therapy) is widely used for the treatment of patients with metastatic breast tumours overexpressing HER2. But the exon-spliced variant of HER2 shows poor recognition for the monoclonal antibody trastuzumab, resulting in drug resistance [10–12]. In addition, protein isoforms are increasingly being recognized as diagnostic biomarkers for a range of diseases, including various cancers [13–15]. This growing recognition underscores the significance of uncovering structural and expression-level differences in protein isoforms. Such analysis provides significant insights across various domains, including diagnostics, prognostics, and therapeutics, to drive global efforts in exploring disease-specific sequence, structural, and functional divergences.

Among the factors that influence protein function, post-translational modifications (PTMs) have been illustrated to have significant functional consequences [16]. PTMs are essential for the diversity of cellular functions, including signalling pathways [17], protein stability [18,19], protein–protein interactions, protein localization [20], and enzymatic activity [21]. They are also linked to several diseases, such as cancer [22], cardiovascular [23], renal [24], neurological [25], and metabolic disorders [26,27]. The rapid advancement of technologies in structural biology, proteomics, and pharmacology has made PTMs as key targets in disease research. This focus is also extendable to protein isoforms, as the isoform-specific diversity of proteins can modify the regulation and overall function of a protein [28–30]. Notably, the interplay between alternative splicing and PTMs adds an additional layer of complexity to protein regulation, as isoform variation can lead to the loss or gain of PTM sites, resulting in altered protein function. Such protein isoform-level variations are also significant to transmembrane (TM) proteins, which constitute over 20% of the human proteome and include enzymes, transporters, ion channels, and receptors of various physiological and nonphysiological ligands. They are critical for various cellular functions, including ion/molecule transport, cell adhesion, ligand–receptor interaction, catalysis of molecular reactions in biological membranes, and more generally, mediating cell–cell interactions and intracellular signal transduction [31,32]. Many of them serve as key drug targets due to their accessibility and regulatory roles in cellular signalling. Alternative splicing in these proteins can also lead in distinct TM topology attributable to the variations in the number or amino acid position of transmembrane regions (TMRs), extracellular loops, or cytoplasmic domains [33–35]. This in turn affects ligand binding, protein interactions, ion selectivity, and signalling [36].

Accounting for these features, protein isoforms may demonstrate functional divergence due to variant constitution of their domains and intrinsically disordered regions (IDRs). Domains are typically highly conserved segments of the protein sequence that confer defined structural or functional roles [37]. Although IDRs lack a fixed three-dimensional structure, they are significant for diverse cellular processes, including signal transduction [38], transcriptional and translational regulation [39], RNA processing, cell cycle control, and small molecule storage [40,41]. Isoform-specific variation in these regions may alter protein stability, interaction networks, and functional outcomes, underscoring the need to consider domain and IDR differences when studying protein function at the isoform level. Although there are several tools to analyse the IDR in proteins, analyses at the isoform level remain largely unexplored [42–45].

Initial large-scale efforts were made to develop resources and studies that facilitate comprehensive analyses of protein expression and diversity [46,47]. In this context, the recent establishment of various splicing variant databases, such as OncoSplicing [48], AScancerAtlas [49], APPRIS [50], ISOexpresso [51], FLIBase [52], ISOdb [53], and Aspdb [54], has yielded comprehensive information pertaining to isoforms.. However, the resources provide comprehensive annotation of protein-level features including TM topology, domain organization, IDRs, and PTMs in a single platform remain limited. Therefore, to meet these demands, we developed IsoProDB (https://ciods.in/isoprodb), an integrated unified database for the analysis of similarities and differences of protein isoforms in humans. This database includes protein isoforms from primary resources (RefSeq and UniProtKB) and provides information on PTMs, protein domain regions including isoform-specific IDRs, predicted topology of TM proteins, and a map of known genetic variants of protein isoforms along with their conservation across isoforms. Currently, IsoProDB covers 110 149 isoforms corresponding to 20 536 protein-coding genes from RefSeq and UniProtKB, offering successful matches for 46 547 protein isoforms across these resources. IsoProDB is accessible to researchers from diverse backgrounds, enabling them to explore and compare protein isoforms with ease, regardless of their bioinformatics expertise.

## Materials and methods

### Data resources

Experimentally validated and predicted protein isoforms corresponding to 20 536 protein-coding genes and the gene information were downloaded from the RefSeq (Release-230) and UniProtKB (Release 2025_02) databases. The RefSeq dataset contained 198 024 transcripts with 101 440 protein isoforms, while the UniProtKB dataset contained 227 203, including 42 534 reviewed (Swiss-Prot) and 184 668 unreviewed (TrEMBL) protein isoform sequences. Mapping between the RefSeq and UniProt accessions of these protein isoforms was performed using in-house Python scripts based on exact full length sequence matching. RefSeq proteins with 100% sequence identity across the entire sequence length to a UniProtKB entry were considered valid matches. Initially, all RefSeq protein isoforms were mapped against 42 534 reviewed (Swiss-Prot) protein isoforms, and the remaining unmapped RefSeq protein isoforms were subsequently compared against 184 668 unreviewed (TrEMBL) UniProtKB protein isoforms. This mapping resulted in 46 547 protein isoforms shared between RefSeq and UniProtKB, and 8710 and 56 340 unique protein isoforms were identified exclusively in UniProtKB (reviewed entries) and RefSeq, respectively. As a result, 110 149 protein isoforms corresponding to 20 536 genes, including all reviewed UniProtKB protein isoforms and only the mapped unreviewed (TrEMBL) protein isoforms from UniProtKB, as well as all RefSeq protein isoforms, were included in IsoProDB. Notably, in the RefSeq database, multiple accessions are provided to identical proteins depending on the different transcript IDs. These identical proteins are grouped by ‘isoform column’ in transcript table provided by RefSeq to remove redundancy and only one representative protein accession is displayed in the PTMs, domain, sequence variant, and topology sections of the web interface.

#### Topology of TM proteins

Although TM proteins account for over 20% of the human proteome, difficulty in determining their structures has resulted in the deposition of a limited number of TM protein structures in the Protein Data Bank [55,56]. To attain the topology of all TM proteins, we used DeepTMHMM v1.0.1 [57], a deep learning-based protein language model that is based upon a deep learning encoder–decoder sequence-to-sequence model that takes a protein sequence as input and outputs the corresponding per-residue sequence of labels. The per-residue labels are signal peptide (S), inside cell/cytosol (I), alpha membrane (M), beta membrane (B), periplasm (P) and outside cell/lumen of ER/Golgi/lysosomes (O). The sequence of residue labels defines the topology of the protein. To analyse the topology, we input the FASTA sequence of all 110 149 protein isoforms in the tool, and the analysis resulted in 22 049 protein isoforms corresponding to 5098 genes as TM proteins, and 16 732 genes were found to have zero TMRs, i.e. nontransmembrane protein isoforms. 99.92% of these proteins have alpha helix structures, and 14 protein isoforms are found to have beta sheets. Among 5098 genes, 1294 are predicted to have at least one nontransmembrane protein isoform along with TM protein isoforms. There are 11 118 protein isoforms corresponding to 1294 genes, in which 3885 are nontransmembrane protein isoforms and 7233 protein isoforms are found to have TMRs. Interestingly, it was noticed that 1230 of the 5098 genes had isoforms with varying numbers of TMRs. The 9098 isoforms with different TMR composition included both multipass and single-pass membrane proteins.

#### PTMs

To collate known PTMs from protein isoforms, PTMs data were downloaded from the dbPTM [58], qPTM [59], and PhosphoSitePlus [60] databases. The data included PTM site, modification type, and protein accessions (UniProt accessions) along with their corresponding references. All PTM sites corresponding to each protein isoform retrieved from the databases were uniformly and nonredundantly mapped to the respective protein isoform sequences by matching UniProt accessions. The mapping process further involved cross-verification of the modified amino acid residue positions against the corresponding protein isoform sequences in the database to ensure positional accuracy and data integrity. This resulted in the mapping of 840 734 PTM sites featuring 52 types of PTMs across 31 325 protein isoforms corresponding to 19 124 genes. Phosphorylation was found to be the most observed PTM, mapped to 636 320 sites corresponding to 27 834 protein isoforms, followed by ubiquitination (165 420 sites in 20 338 protein isoforms) and methylation (131 404 sites in 20 275 protein isoforms). Protein H3C4 (P68431) was found to be the highest modified protein (18 PTM types), while 1107 proteins had no known PTMs attributed in any of the databases.

To assess the conservation and uniqueness of PTM sites among protein isoforms, a sequence alignment-based conservation analysis was incorporated. The conservation of sites is analysed by aligning the protein isoform sequences using BioMSA, a JavaScript library that enables local alignment of sequences (DNA or protein) within the browser. After alignment, a nine-amino acid window centred on each PTM site was evaluated to assess sequence conservation across isoforms of the same protein. A PTM site was considered conserved if this nine-amino acid region was aligned and preserved across all protein isoforms. Along with the conservation, the alignment provides sites which are specific to protein isoforms.

#### Disordered regions and functional domains

Even though there are various tools to explore the IDRs in proteins, the variation of these across the isoforms is unexplored. Moreover, the information of experimentally validated IDRs in proteins is limited. The disordered regions in protein isoforms were predicted using InterProScan, a standalone tool developed by InterPro [61]. InterProScan uses MoBiDB, a comprehensive database that provides annotations and predictions of IDRs in proteins from protein sequences. The analysis resulted in the prediction of IDRs in 32 389 protein isoforms belonging to 11 748 genes. Similarly, domain details of protein isoforms were analysed using InterProScan, which integrates FASTA sequence-driven domain architecture data from six different databases, including Pfam [62], Conserved Domains Database (CDD) [63], PROSITE [64], CATH-Gene3D [65], PRINTS [66], and SMART [67]. As a result, IsoProDB contains comprehensive domain architecture for 40 205 protein isoforms corresponding to 14 261 genes, which is highly useful for comparing isoform-specific domain architecture from different domain resources and for comparative analysis with other isoforms of the same protein.

#### Clinically relevant sequence variants among isoforms

Sequence variation among protein isoforms plays a critical role in defining their structural/functional diversity and similarity, making it essential to analyse isoform-specific sequences when investigating protein isoform characteristics. To enable analysis of the impact of sequence variation on protein isoforms, variant data were obtained from the ClinVar [68] and gNOMAD [69] databases. The data included variation in the transcript, subsequent change in the protein sequence, variant type, clinical significance, transcript identifier, and protein accession from both the databases and removed redundancy in data. This variance data was mapped with protein isoforms by the RefSeq protein accession, resulting in the catalogue of 65 039 protein isoforms corresponding to 18 498 genes with sequence variants.

### Technological framework for development of IsoProDB

**Figure 1:**
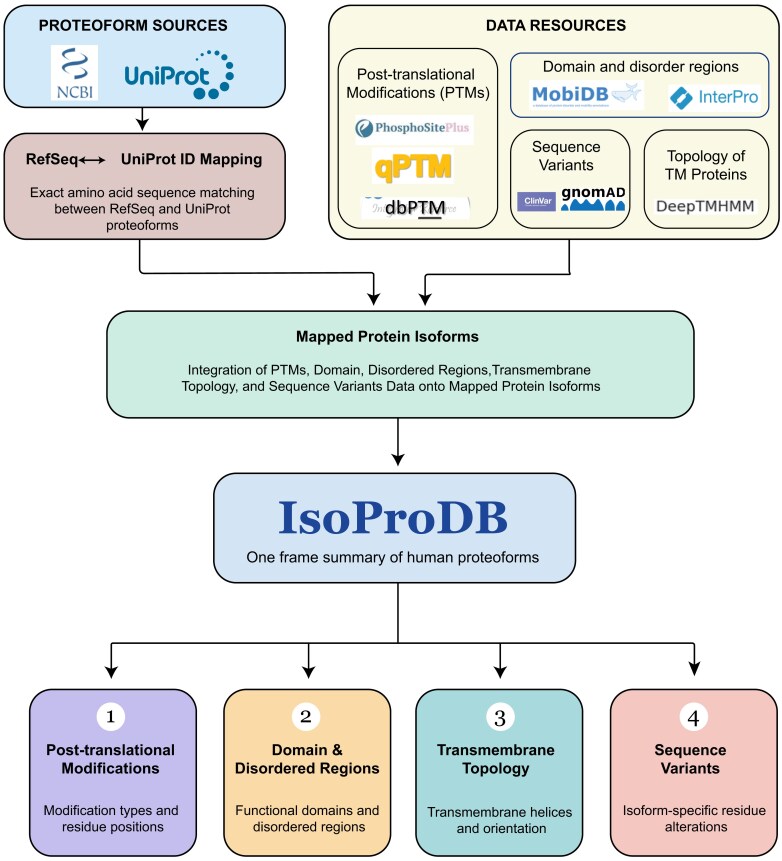
The schematic illustrates the methods and resources used for the development of IsoProDB.

The IsoProDB database was developed using a Django framework running in a Docker container in the backend, and the frontend was built with React.js and styled using Tailwind CSS. My Structured Query Language (MySQL) database management system was utilized for the storage and management of data, providing robust and scalable solutions for data management. The database, along with its packages makes storing and retrieving data simple, fast, and useful for the application. All codes and scripts used for data analysis and integration in this study are available at the GitHub repository https://github.com/sree-pathappillil/IsoProtDB. A schematic overview of the methodology is provided in Fig. 1.

## Results and discussion

### The web interface

**Figure 2:**
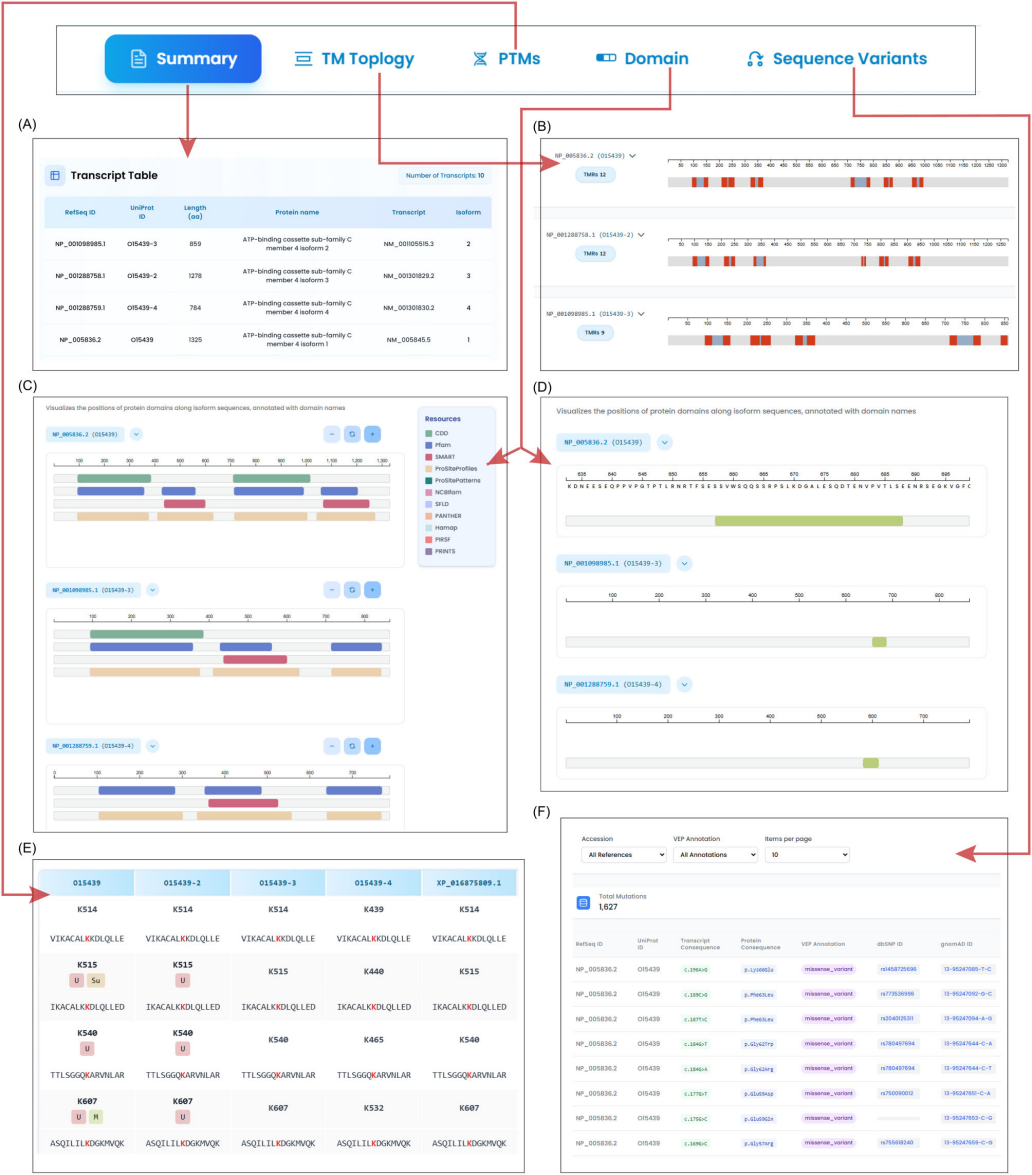
The figure illustrates the isoform-specific exploration of ABCC4. The search bar navigates to the summary page, where (A) the transcript table lists all the transcripts and isoforms of ABCC4 along with the protein information. (B) Topology section shows the number of TMRs in all protein isoforms along with their range in protein sequence. (C) The domain details of three protein isoforms, with parent databases that contribute to domain details are highlighted in different colours. (D) The IDRs of three protein isoforms are shown in the disordered region section within the domain tab. (E) The site table lists all the modified sites in three protein isoforms. The site is labelled with all the reported modifications along with a sequence window. The aligned sites are given in a row for each site. (F) All the variants in the protein isoforms are listed in the table along with the transcript and protein level changes of ABCC4.

IsoProDB is an integrative platform developed to explore the diversity of human protein isoforms. It uniquely aligns various protein isoforms from primary resources and enables cross-sequence visualization with integrated features such as protein domains, IDRs, sequence variants, TM topology, and over 52 PTMs mapped across isoforms. IsoProDB currently supports the interactive visualization and comparative analysis of sequence, structure, and function for 110 149 protein isoforms derived from 20 536 human protein-coding genes in humans. The web interface is organized into distinct sections that enable comparative analysis of protein isoforms in terms of PTMs, domains, and IDRs, as well as sequence variants and TM topology. Figure 2 shows an overview of the IsoProDB user interface using the example gene ABCC4.

The Home page provides the statistics and overview of the database along with a search bar (Fig. S1). Users can browse IsoProDB by gene symbol (e.g. ABCC4 and BRCA1) through search bar on the homepage, which directs to the protein summary page, where the information of the query gene is provided with a list of protein isoforms along with the transcripts as a table (Fig. 2A; Fig. S2A). The page also provides the sequence alignment of all isoforms aimed at analysing the regions that are conserved and those that vary across isoforms (Fig. S2B). Along with the summary page, the user can access all five sections in the database, including PTMs, TM topology, domain and sequence variants, and each of these sections provides information and visualization of unique protein isoforms of the gene of interest. Even though the summary page lists all the transcripts and isoforms of the queried gene, only unique isoforms are represented in the rest of the tabs.

Adjacent to the protein summary, the TM Topology section provides the predicted TM topology for each protein isoform of the query gene. The accompanying table (Fig. S3) and visualization support comparative analysis across protein isoforms, allowing users to easily identify similarities and differences in the number and range of TMRs among them (Fig. 2B). This section also includes results for the nontransmembrane proteins in such a way that they are either inside or outside the membrane.

**Figure 3:**
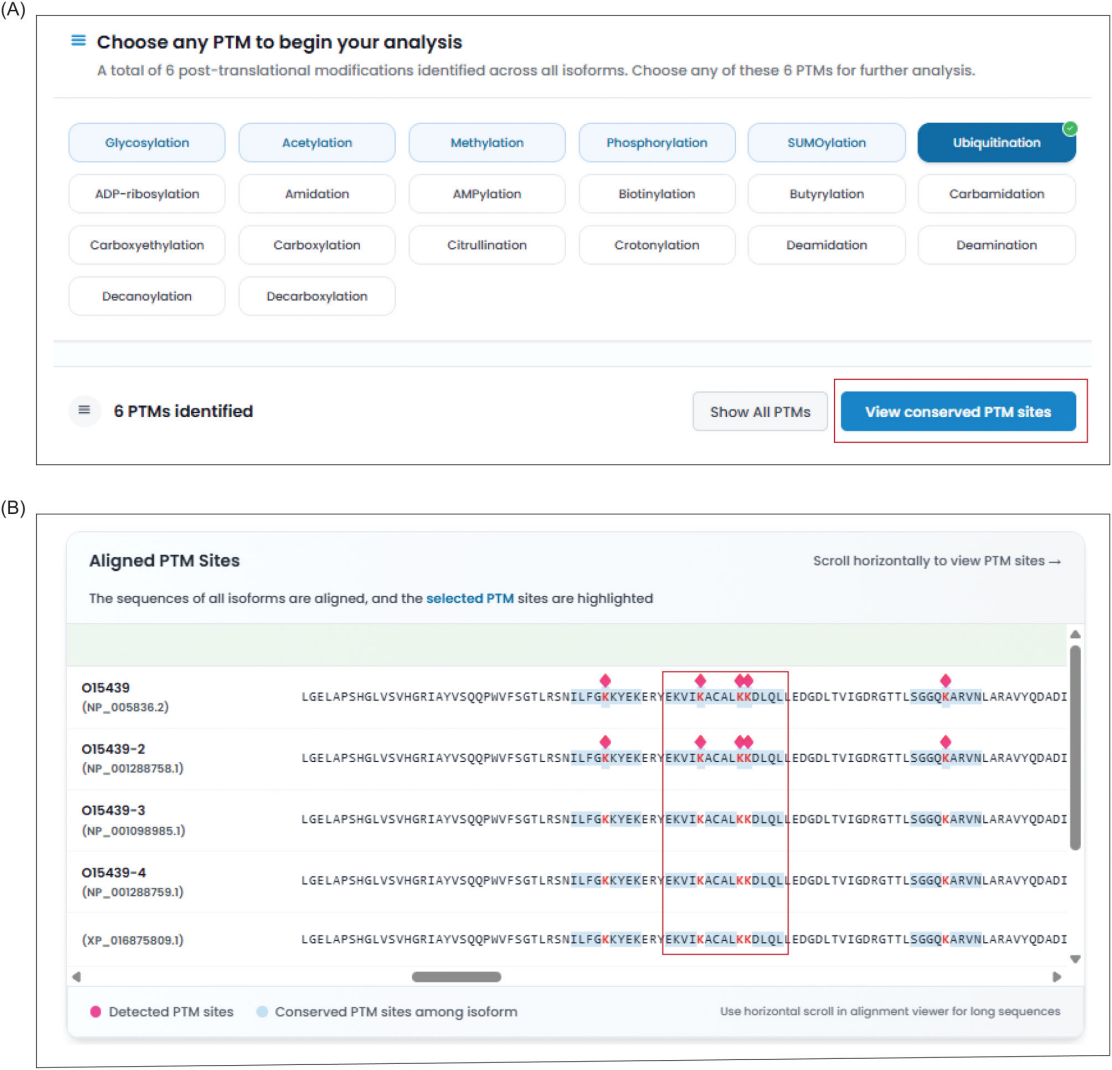
The figure illustrates the conservation analysis of PTM sites in ABCC4. (A) The ABCC4 is reported with a total of five PTMs, and the user can select any of them for conservation analysis. The selection of ubiquitination lists the number of reported ubiquitinated sites across the protein isoforms of ABCC4. (B) The conservation analysis button shows all the conserved ubiquitinated sites among the isoforms and highlights them in blue colour within the alignment viewer in all isoforms. The diamond symbol indicates the reported ubiquitinated sites in protein isoforms.

The PTMs section in IsoProDB includes 52 types of PTMs detected in 20 536 protein-coding genes. The query for each gene highlights only the reported PTMs in these 52 types of protein isoforms and allows users to choose any of them for analysis. Once selected, the sites that are reported with the selected PTM type are displayed on the corresponding protein isoform within the alignment view section, alongside a detailed data table. The section also provides a site conservation analysis, which identifies regions with PTM sites conserved across protein isoforms, and this helps to infer the potential occurrence of PTMs in protein isoforms, where these sites have not yet been experimentally detected (Fig. 3). The site table lists all reported PTMs across protein isoforms and shows how specific they are to each protein isoform (Fig. 2E). In this context, specificity refers to whether a modified site is unique to a specific protein isoform or conserved across various isoforms, as determined by the sequence alignment of all protein isoforms. This, along with interactive visualizations, enables a clear and effective comparative analysis of selected PTMs across different isoforms.

The Domain section integrates both protein domain and IDR information along with the visualization and data table for all the protein isoforms of the queried gene (Fig. 2C and D). The data table includes domain names, amino acid ranges, and corresponding references to parent databases for more information (Figs S4 and S5). This display helps in understanding domain loss or gain, isoform-specific structural flexibility, and potential regulatory regions that contribute to the functional diversity of protein isoforms. Following the domain, the sequence variant section integrates variant information from the gnomAD and ClinVar databases. The page provides information on the variants, including transcript and protein-level consequences and identifiers, along with entry identifiers from the reference database (Fig. 2F). Moreover, the user can filter the protein isoforms and variant types in the results. This section is designed to help users understand how genetic variation may affect different isoforms, enabling insights into isoform-specific impacts of variants and their potential clinical relevance. Moreover, a graphical representation is provided in the variant section, in addition to the existing tabular data, to improve clarity and usability. Users can download the data corresponding to the queried gene in each section of the database. Furthermore, the information on genes and isoforms in IsoProDB is accessible through the download option in the database. This improves data transparency and enables users to better explore and analyse IsoProDB data. The Help page and FAQ section provide additional information to guide users when accessing and using the database. For further queries, users can reach out to the team through the Contact page.

### Intended functionality and user benefits

IsoProDB is an integrative and user-friendly visualizable platform developed to explore the diverse protein isoforms in humans, particularly considering that their annotation often varies across primary resources and many of them remain unintegrated across resources. Towards this, IsoProDB aligns diverse protein isoforms from UniProtKB and RefSeq, providing mapping between accessions from both databases. It focuses on integrating protein features, such as domain architecture, PTMs, IDRs, TM topology, and sequence variants at the protein isoform level, providing a consolidated view of these attributes within a single framework. Recent studies underscore that variation in the number of TMRs among protein isoforms is a significant factor that deserves greater attention, as it can lead to distinct patterns of tissue-specific expression, subcellular localization, and functional divergence. Extending this concept, vascular endothelial growth factor receptor 1 (VEGFR1/FLT1) also exists as TM and soluble isoforms, illustrating how isoform diversity can have diagnostic relevance. The soluble VEGFR1 (sFLT1) isoforms serve as clinical biomarkers, as their quantification is used to determine the sFLT1: PlGF ratio, a diagnostic indicator that predicts the absence of pre-eclampsia. Although the antibodies used for this assay recognize all VEGFR1 isoforms, the test effectively measures only the soluble FLT1 isoforms since the others are TM forms (Fig. S6) [15, 29,70]. Considering these, the TM topology in IsoProDB offers new perspectives on the concept that TMR-based isoform diversity should be recognized as a key factor in understanding protein function at the isoform level and should be more broadly discussed in the scientific community. IsoProDB acts as a platform that gives information about the TMRs of all protein isoforms, allowing users to analyse nontransmembrane and transmembrane isoforms, variations in the TMR number, etc., of the proteins of interest. In a similar vein, PTMs show notable differences across protein isoforms, adding another layer of functional complexity. For example, STAT3 and its phosphorylated sites have been a promising target in cancer treatments for years, and Y705 is one of them [71–75]. In our analysis, it is found that Y705 is conserved across all 16 STAT3 protein isoforms except NP_001371917.1. Phosphorylation at this site has been reported in only three isoforms, despite its conservation in 16 protein isoforms (Fig. S7). Similarly, in hepatocellular carcinoma (HCC), the isoforms of spleen tyrosine kinase, SYK(L) and SYK(S), exhibit distinct expression patterns and functional roles. SYK(L) expression is generally downregulated in HCC tumour samples compared to normal liver tissue, whereas SYK(S) levels are elevated. This differential regulation is linked to CHK1-mediated phosphorylation, which promotes proteasomal degradation of SYK(L) in HCC [76]. The phosphorylation site (S295) targeted by CHK1 is located within the spliced (DEL) region present only in SYK(L) but absent in SYK(S); consequently, elevated CHK1 levels lead to the selective loss of the full-length SYK(L) without affecting SYK(S) expression (Fig. S8). Functionally, SYK(S) enhances cellular invasion, whereas SYK(L) suppresses metastasis in HCC [77]. The PTM section in IsoProDB allows users to explore and enhance their understanding of patterns of conservation and specificity of reported PTM sites among the protein isoforms, which contributes to a better grasp of protein function, regulation, and potential therapeutic or biomarker applications. Beyond PTMs and TM topology, IsoProDB provides a quick overview of protein isoforms in terms of domain and IDR, as well as sequence variants. Moreover, the data visualization in each section is intended to provide a comparative analysis across protein isoforms for the quick understanding of differences and similarities across protein isoforms. Beyond comparative analysis, IsoProDB enables researchers to investigate the specificity or conservation of particular sites among isoforms and to explore their functional relevance in terms of domains, IDRs, PTMs, and clinical significance associated with sequence variants, all within a single platform.

### Comparison of IsoProDB with other databases

The development of various isoform databases has emerged in recent years, providing various information regarding the protein isoforms, such as OncoSplicing [48], AScancerAtlas [49], FLIBase [52], ISOdb [53], CanIsoNet [78], APPRIS [50], and ASpdb [54]. Most of these resources focus on specific diseases like cancer. OncoSplicing and AScancerAtlas provide information on splicing events in cancer, while FLIBase characterizes and catalogues full-length isoforms using long-read RNA sequencing techniques. In contrast, CanIsoNet focuses on disease-specific isoform interaction networks in humans. APPRIS annotates and identifies principal splice isoforms of protein-coding genes by integrating structural, functional, and domain-based information.

Moreover, while ASpdb provides structural analysis and explores the relationship between splicing variants, diseases, and drugs in humans and APPRIS annotates splice isoforms with protein structure, domains, and TM helices, no other resource brings together important features such as TM topology, domain composition, IDRs, PTMs, and clinical variants in one database. IsoProDB aims to provide detailed information on these features for all listed unique protein isoforms of a queried gene through a structured, unified platform. Interestingly, most of these databases provide information at the transcript level rather than at the protein level, whereas IsoProDB characterizes all protein isoforms derived from the RefSeq and UniProtKB databases. IsoProDB covers 110 149 isoforms corresponding to 20 536 protein-coding genes and successfully matches 46 547 protein isoforms across these resources. This comprehensive integration and unique coverage position IsoProDB as a valuable resource for in-depth isoform-level protein analysis, broadens the scope of existing resources. The comparison of IsoProDB between the existing isoform databases are provided in Table S1.

### Future development and maintenance

IsoProDB addresses extensive isoform-level data. However, there are a few limitations that anticipate updating in the future. First, IsoProDB provides PTM conservation among isoforms by considering primary sequence alignment due to the limited availability of well-defined tertiary structures. This limitation restricts our ability to account for structural context when evaluating the functional conservation of PTM sites. We intend to integrate tertiary structural information in the future, facilitating a more precise and spatially informed comprehension of PTM conservation across protein isoforms. Additionally, the current version of IsoProDB does not incorporate interactors of proteins at the isoform level. The inclusion of isoform-specific interaction networks in future updates would provide deeper insights into the distinct functional roles and regulatory mechanisms of individual protein isoforms. Furthermore, although clinical relevance has been partially addressed through the integration of sequence variant data, direct associations between specific isoforms and disease phenotypes are not yet established. We intend to incorporate such information as an additional data layer to further support disease-focused and clinical research applications.

## Conclusion

IsoProDB serves as an integrative platform for the understanding and analysis of protein isoform diversity in humans. In recent years, protein isoform characterization has gained considerable attention since differences among isoforms can have significant functional implications in diverse areas, particularly therapeutics. The regulatory mechanism, cellular localization, functional properties of protein isoforms in terms of PTMs, domain, and IDRs, and the TM topology are not explored in the existing databases, making IsoProDB unique. And these characterizations at the protein level of all the isoforms, regardless of their expression in specific diseases, establish IsoProDB as a reference database for global users. Moreover, the inclusion of all protein isoforms from UniProtKB and RefSeq and the cross-reference between the databases provides a user-friendly unified platform for the analysis. Although IsoProDB currently provides protein isoform information at the sequence level, future updates will extend its coverage to include structural-level details. Together, IsoProDB addresses a critical gap in the field by providing a powerful, isoform-centric platform, serving as a foundational resource for future discoveries in genomics, structural biology, and translational research.

## Supplementary Material

baag015_Supplemental_File

## Data Availability

All codes and scripts used for data analysis and integration in this study are available at the GitHub repository https://github.com/sree-pathappillil/IsoProtDB. The datasets used and/or analysed during the current study are available from the corresponding author on reasonable request.
