# Supplementary material for: IsoProDB: an integrated map of human protein isoforms for accelerated research
Source: Database (Oxford). 2026 Mar 26;2026:baag015. doi: 10.1093/database/baag015 (PMC13019292; doi:10.1093/database/baag015)
Supplement: baag015_Supplemental_File [file baag015_supplemental_file.docx]

**SUPPLEMENTAL FIGURES AND TABLES**

**
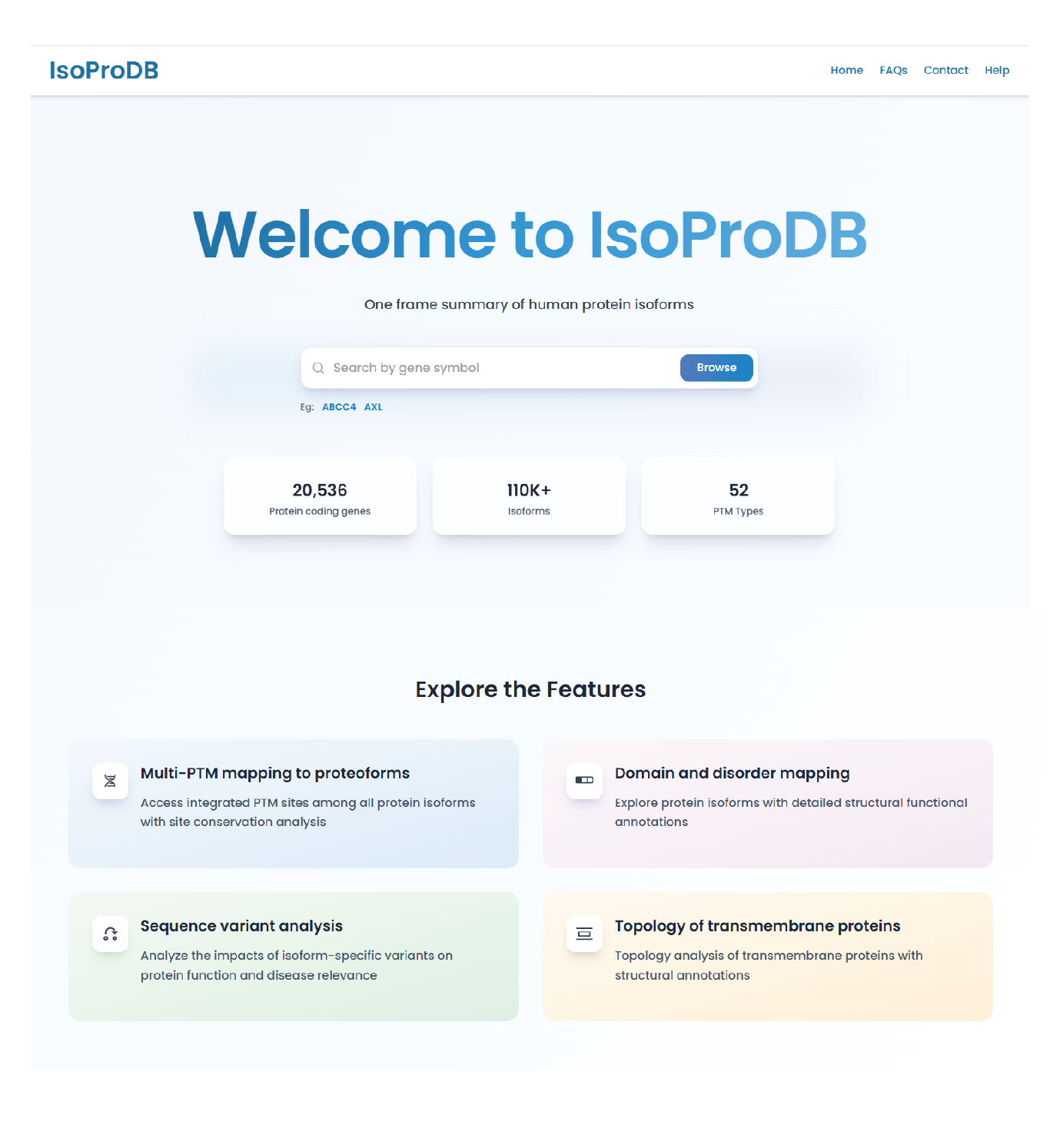
**

**Supplementary Fig. S1. The Home page of IsoProDB.** The home page includes the search bar along with summary of the database. The user can query the database using the gene symbol and explore the features provided. The examples are provided along with the search bar.

**
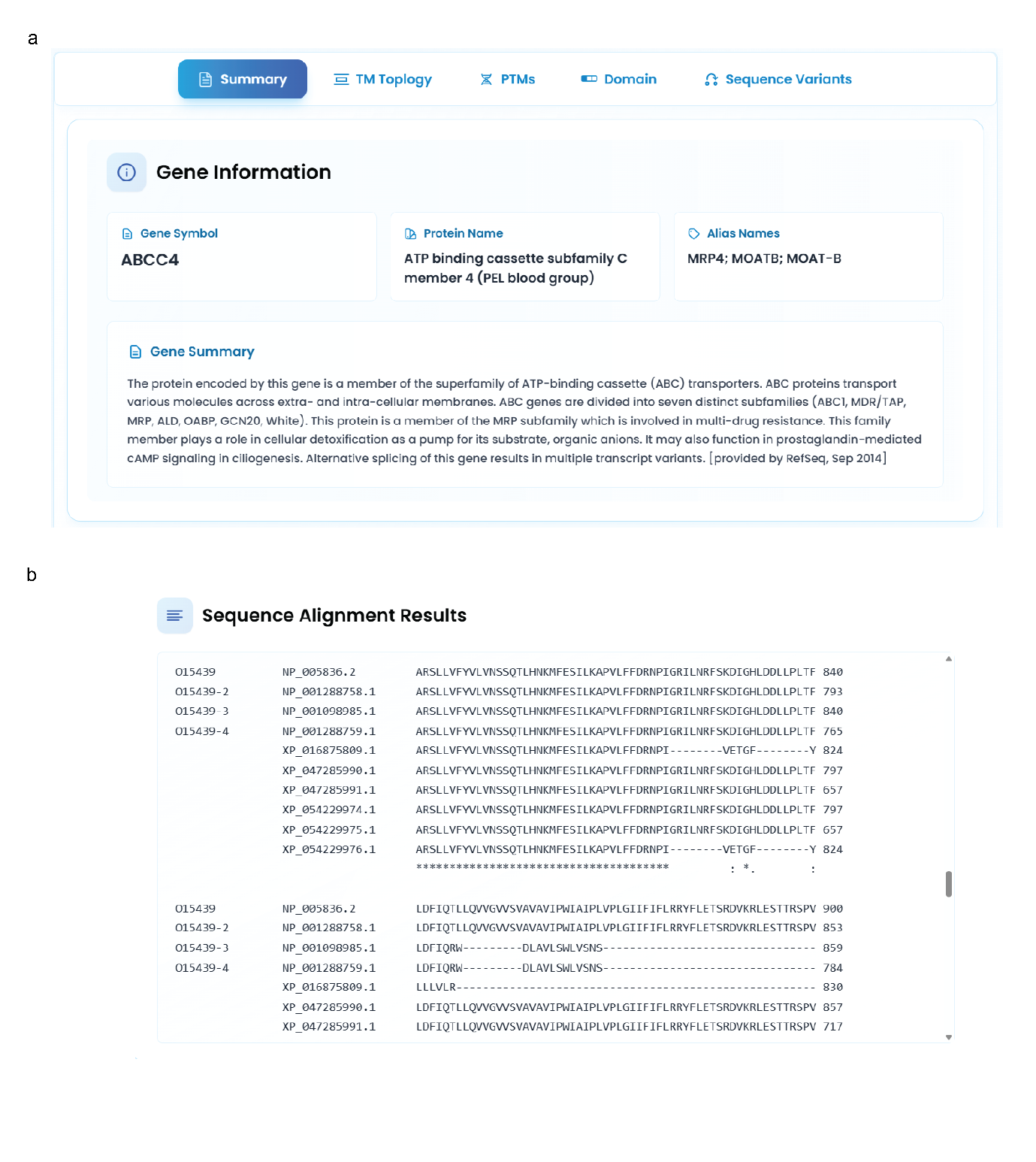
**

**Supplementary Fig. S2. The summary page of ABCC4. A)** The summary page included information about the protein. **B)** The result of sequence alignment of all proteoforms using BioMSA


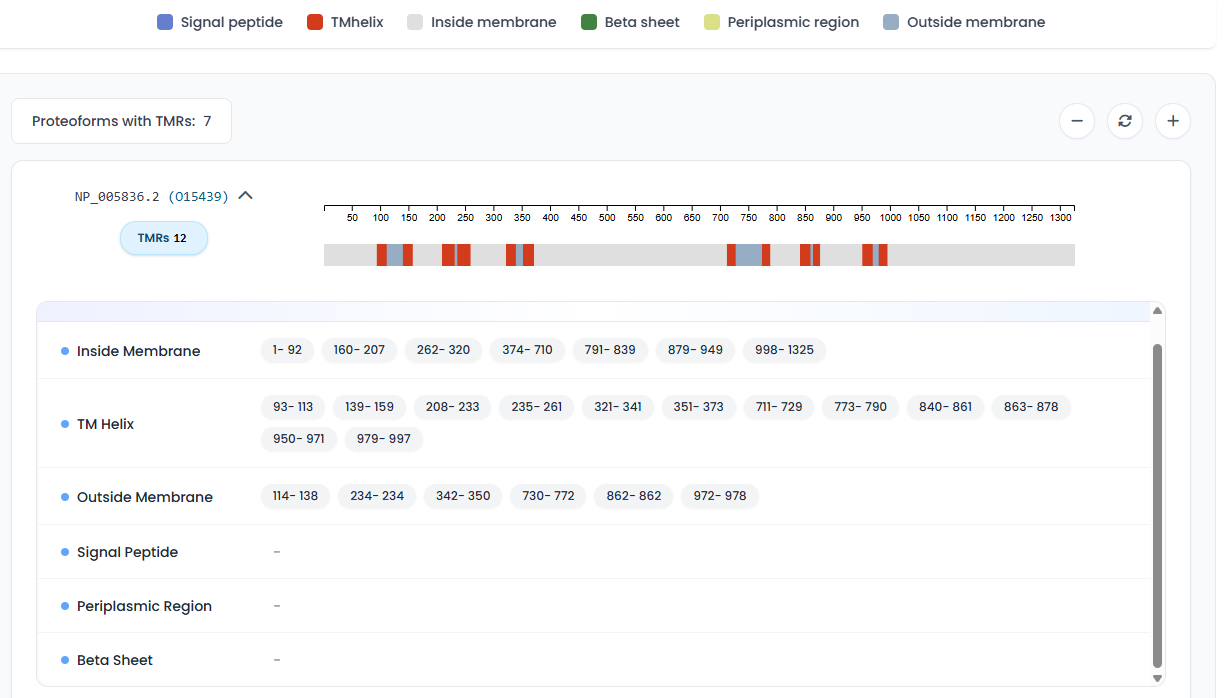


**Supplementary Fig. S3. The transmembrane topology of proteoform NP_005836.2 of ABCC4.** The ABCC4 have 7 transmembrane proteoforms. The proteoform NP_005836.2 contain 12 transmembrane regions. The TM regions and its range are visualized and represented in the table also.


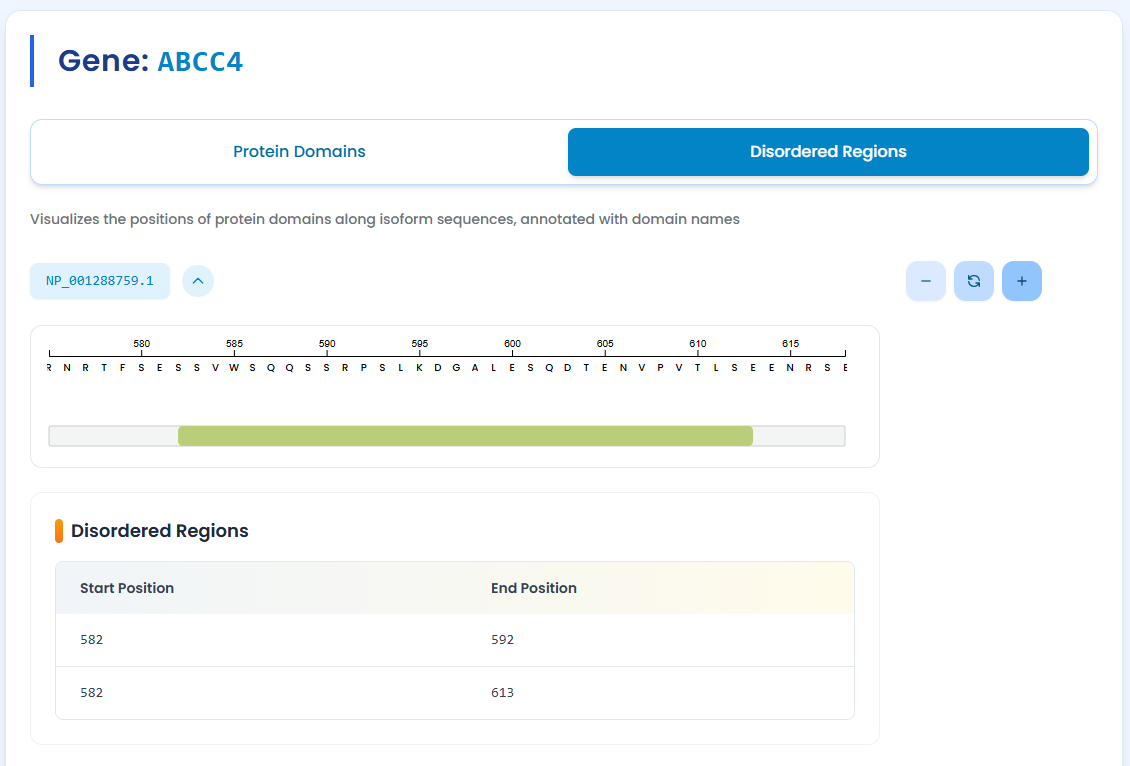


**Supplementary Fig. S4. The information of domain of proteoform NP_001288759.1 of ABCC4.** The table shows the domain name, its ranger over the amino acid sequence and the name of the resource database from InterPro. The table also provides the link to the source database.


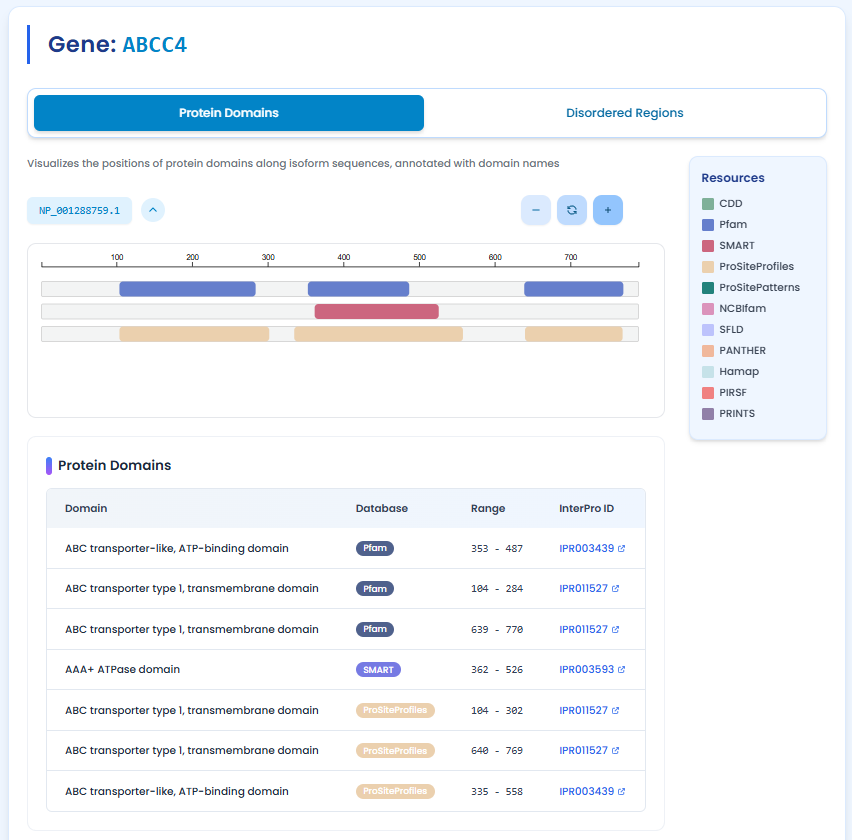


**Supplementary Fig. S5. The information of intrinsically disordered regions of proteoform NP_001288759.1 of ABCC4.** The table shows the range of intrinsically disordered regions over the protein sequence along the with the visualisation.


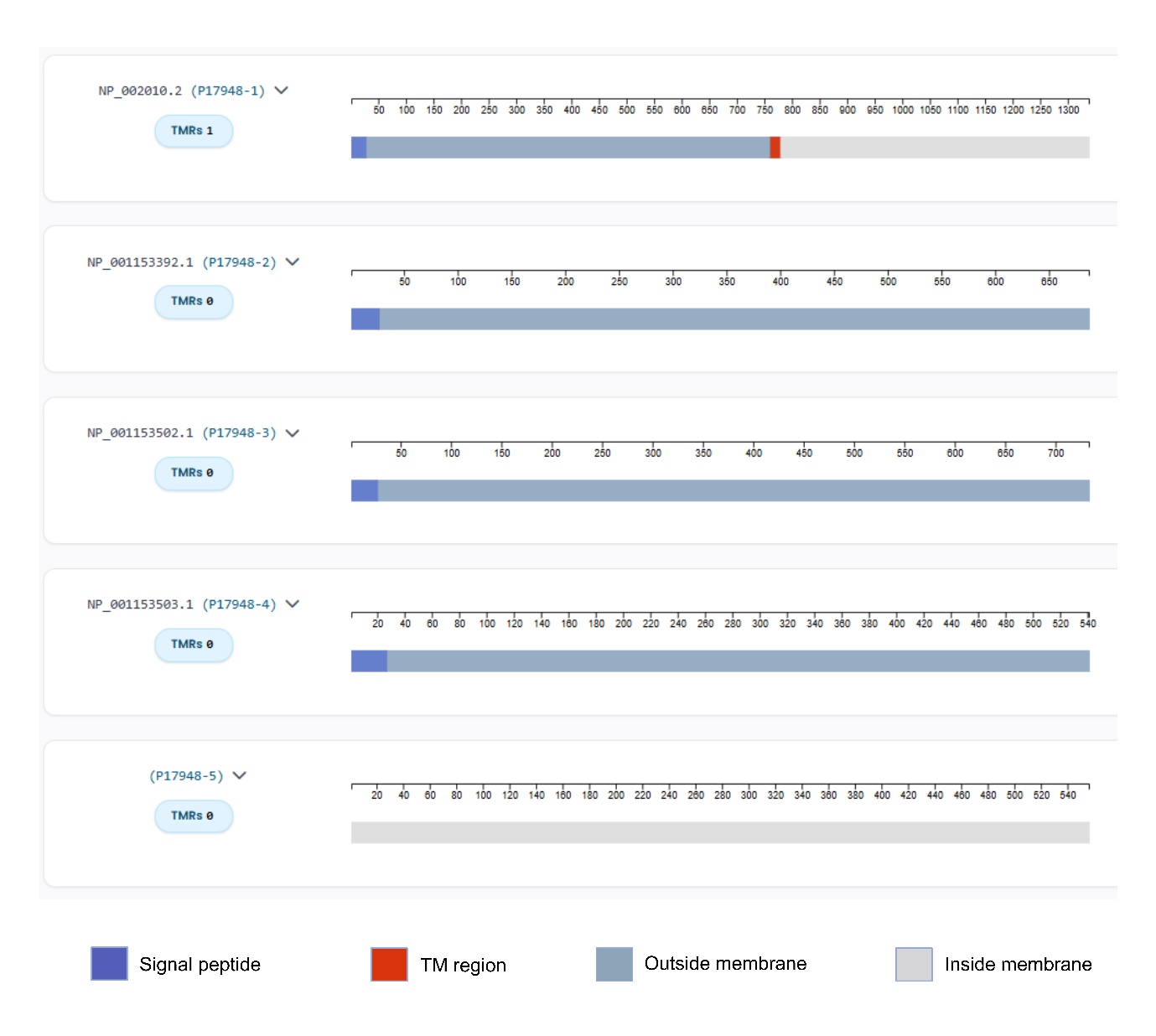


**Supplementary Fig. S6. The protein isoforms of FLT1 display different transmembrane topology.** Among the 5 protein isoforms of FLT1, NP_002010.2 harbours a transmembrane domain. The NP_001153392.1, NP_001153502.1 and NP_001153503.1 are secreted proteins, while P17948-5 are inside the membrane.


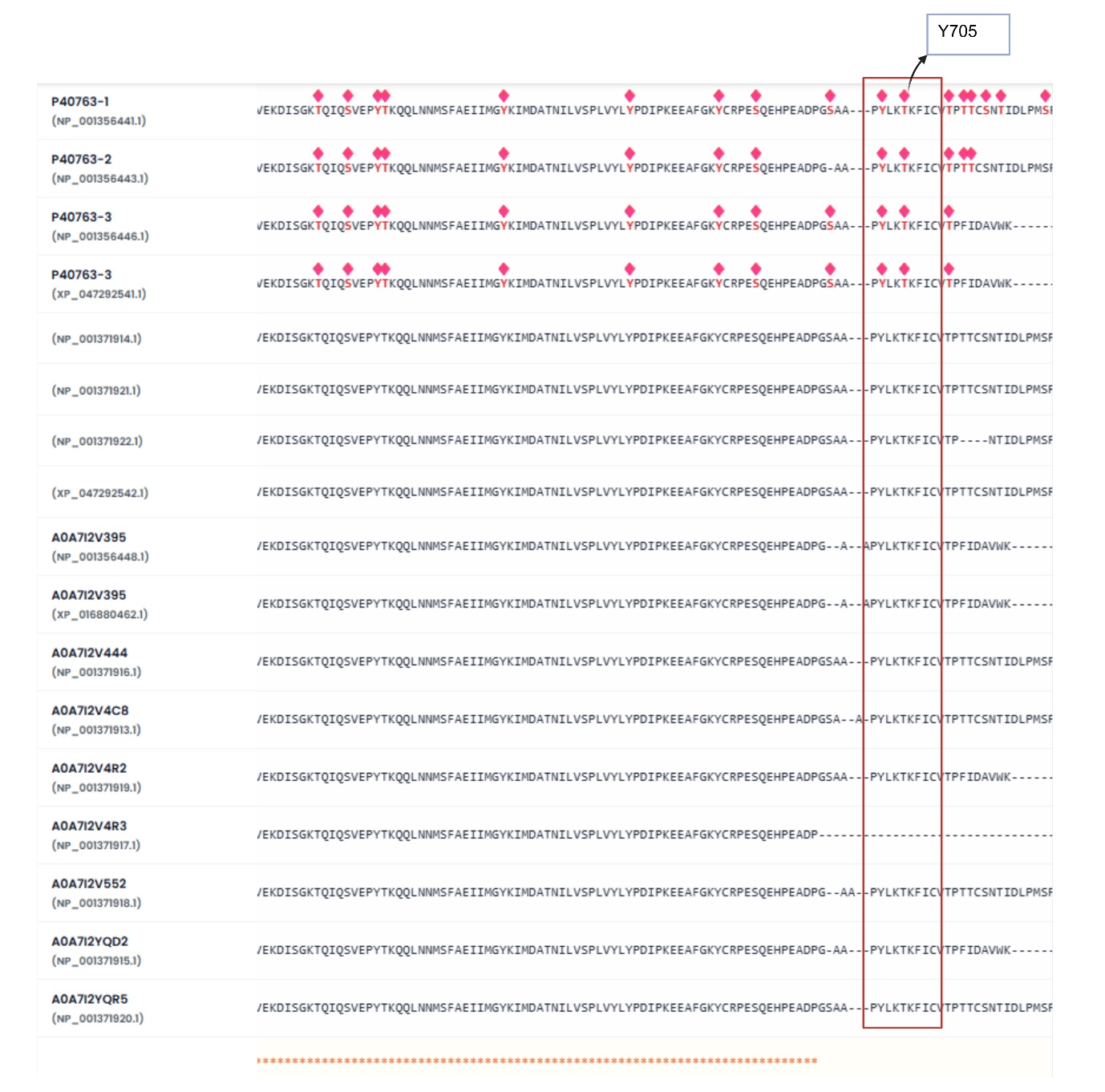


**Supplementary Fig. S7. The conservation of Y705 across the protein isoforms of STAT3.** The sequence alignment of all isoforms of STAT3 wherein Y705 is conserved in all the isoforms except for NP_001371917.1.


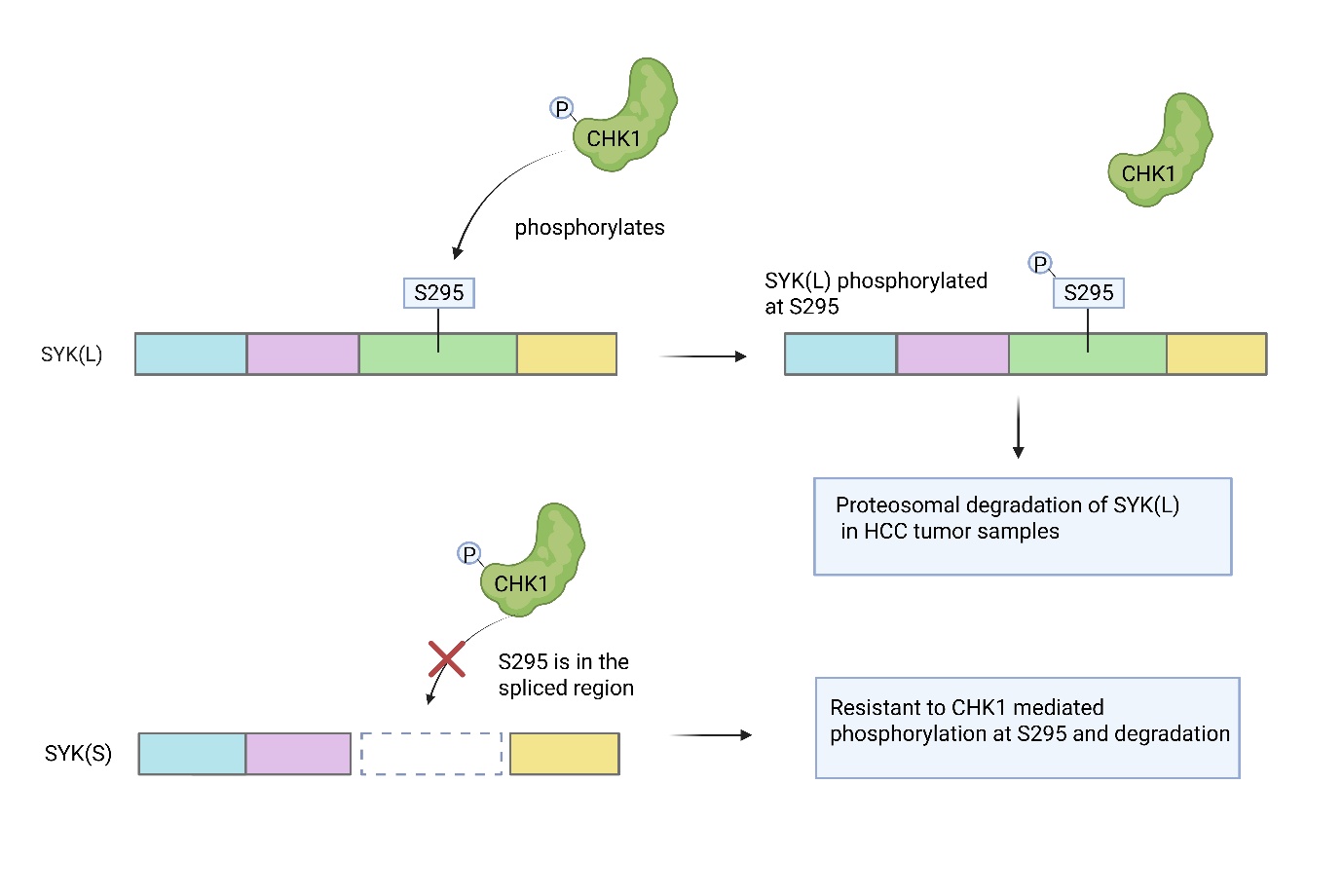


**Supplementary Fig. S8. Phosphorylation at S295 in isoforms of SYK.** CHK1 phosphorylates SYK(L) at Ser295, leading to proteasomal degradation in HCC cells, whereas the isoform, SYK(S) lacks this site and is resistant to CHK1-dependent degradation.

|  | **FLIBase** | **Onco Splicing** | **ASCancer Atlas** | **CanIso Net** | **ASpdb** | **APPRIS** | **IsoProDB** |
| --- | --- | --- | --- | --- | --- | --- | --- |
| Protein level | No | No | No | No | Yes | Yes | Yes |
| Transcriptslevel | Yes | Yes | Yes | Yes | Yes | Yes | No |
| Disease specific | Yes | Yes | Yes | Yes | No | No | No |
| Post-translational modification | No | No | No | No | No | No | Yes |
| Topology of TM proteins | No | No | No | No | No | Yes | Yes |
| Domain architecture | No | No | No | No | No | Yes | Yes |
| Data Resource | TCGA/ GTEx | TCGA/ GTEx | TCGA/ GTEx/ literature curation | PCAWG/ GTEx | UniProtKB | GEN CODE | UniProt KB/  RefSeq |
| Structural information | No | No | No | No | Yes | Yes | No |

**Supplementary Table S1.** The comparison of IsoProDB between the existing isoform databases.
